# A subcutaneous adipose tissue–liver signalling axis controls hepatic gluconeogenesis

**DOI:** 10.1038/ncomms7047

**Published:** 2015-01-12

**Authors:** Shannon M. Reilly, Maryam Ahmadian, Brian F. Zamarron, Louise Chang, Maeran Uhm, BreAnne Poirier, Xiaoling Peng, Danielle M. Krause, Evgenia Korytnaya, Adam Neidert, Christopher Liddle, Ruth T. Yu, Carey N. Lumeng, Elif A. Oral, Michael Downes, Ronald M. Evans, Alan R. Saltiel

**Affiliations:** 1 Life Sciences Institute, University of Michigan, Ann Arbor, Michigan 48109, USA; 2 Gene Expression Laboratory, Salk Institute for Biological Sciences, La Jolla, California 92037, USA; 3 Program in Immunology, University of Michigan, Ann Arbor, Michigan 48109, USA; 4 Department of Internal Medicine, Metabolism, Endocrine and Diabetes Division, University of Michigan, Ann Arbor, Michigan 48109, USA; 5 Storr Liver Unit, Westmead Millennium Institute and University of Sydney, Westmead Hospital, Westmead, New South Wales 2145, Australia; 6 Department of Pediatrics and Communicable Diseases, University of Michigan, Ann Arbor, Michigan 48109, USA

## Abstract

The search for effective treatments for obesity and its comorbidities is of prime importance. We previously identified IKK-ε and TBK1 as promising therapeutic targets for the treatment of obesity and associated insulin resistance. Here we show that acute inhibition of IKK-ε and TBK1 with amlexanox treatment increases cAMP levels in subcutaneous adipose depots of obese mice, promoting the synthesis and secretion of the cytokine IL-6 from adipocytes and preadipocytes, but not from macrophages. IL-6, in turn, stimulates the phosphorylation of hepatic Stat3 to suppress expression of genes involved in gluconeogenesis, in the process improving glucose handling in obese mice. Preliminary data in a small cohort of obese patients show a similar association. These data support an important role for a subcutaneous adipose tissue–liver axis in mediating the acute metabolic benefits of amlexanox on glucose metabolism, and point to a new therapeutic pathway for type 2 diabetes.

## Abstract (web summary)

The drug amlexanox is known to improve obesity-related metabolic dysfunction in mice. Here the authors show that this effect is mediated by interleukin-6 secreted from subcutaneous adipocytes, which then inhibits gluconeogenesis in the liver by phosphorylating the hepatic transcription factor Stat3.

## Graphical abstract

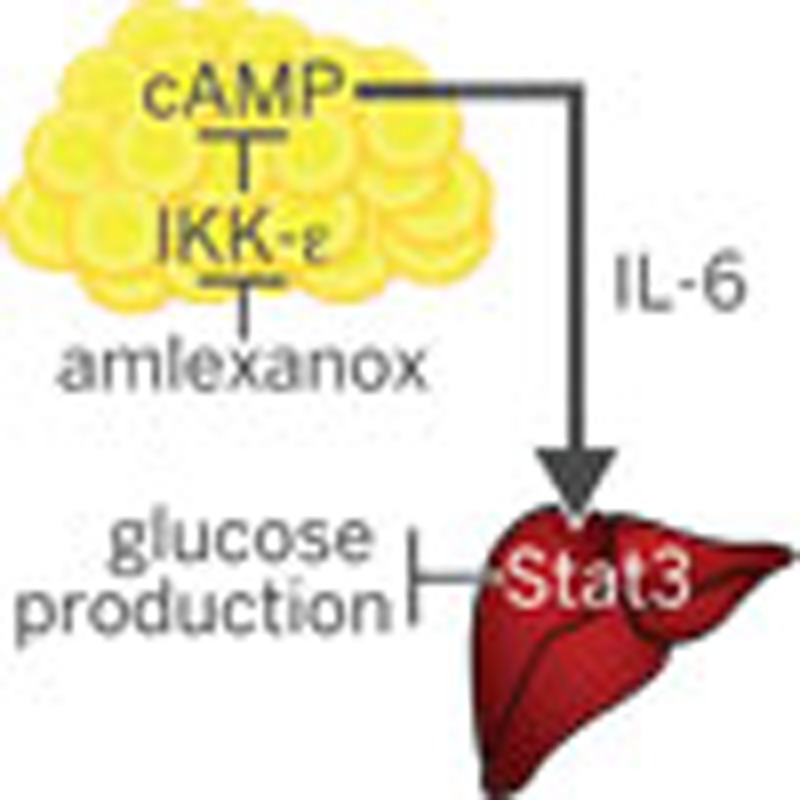

Accumulating evidence indicates that the chronic low-grade inflammation associated with obesity plays an important role in the development of metabolic disease. Obesity induces an increase in the NF-κB pathway^1^,^2^,^3^, which leads to induction of the non-canonical IKKs, IKK-ε and TBK1 in adipose tissue and liver^4^,^5^,^6^,^7^. Once induced, these kinases can initiate a counter-inflammatory programme by phosphorylating and activating phosphodiesterase 3B (PDE-3B) in adipocytes, thus producing reduced levels of cAMP and resistance to stimulation by catecholamines such as epinephrine and norepinephrine^8^. This resulting catecholamine resistance reduces energy expenditure and thermogenesis, and contributes to the development of obesity in humans and mice^8^,^9^,^10^,^11^,^12^. Our previous studies demonstrated that inhibition of IKK-ε/TBK1 with the dual-specificity inhibitor amlexanox improved catecholamine and insulin resistance, and resulted in significant weight loss and reversal of metabolic disease in obese mice, but had no effect on normal-chow fed mice in which the kinases are not elevated^8^,^13^.

While improved catecholamine sensitivity may account for the dramatic weight loss observed after IKK-ε and TBK1 inhibition in obese mice, the mechanism of improved insulin sensitivity and glucose handling remains uncertain. A euglycemic–hyperinsulinemic clamp revealed that the insulin-sensitizing effects of amlexanox were mainly attributed to suppression of hepatic glucose production^13^. Moreover, insulin sensitization was observed prior to significant weight loss in obese mice. However, it was not clear whether the effects of amlexanox in the liver were mediated through direct inhibition of hepatic IKK-ε/TBK1 or another indirect mechanism. Here we describe one indirect pathway by which inhibition of IKK-ε and TBK1 in the subcutaneous adipose tissue affects reduced hepatic glucose production. Specifically, inhibition of IKK-ε and TBK1 by amlexanox stimulates the secretion of interleukin-6 (IL-6) from adipocytes as well as preadipocytes in the subcutaneous adipose tissue via a cAMP/p38-dependent pathway. The resulting increase in serum IL-6 is responsible for the activation of hepatic Stat3, which suppresses expression of *G6pc* to reduce hepatic glucose output.

## Results

### Direct versus indirect effects of amlexanox in the liver

To determine the mechanism by which amlexanox improves hepatic insulin sensitivity, we performed RNA sequencing analysis of hepatic gene expression a few hours after *in vivo* amlexanox treatment, and identified over 1,700 differentially expressed genes (GEO accession code GSE57054). Pathway analysis of these differentially regulated genes revealed that the top two most enriched pathways were the adipocytokine signalling pathway and the Jak/Stat signalling pathway, presumably activated by a tissue-derived ligand (Supplementary Table 1).

### Amlexanox treatment induces IL-6 signalling

**Figure 1:**
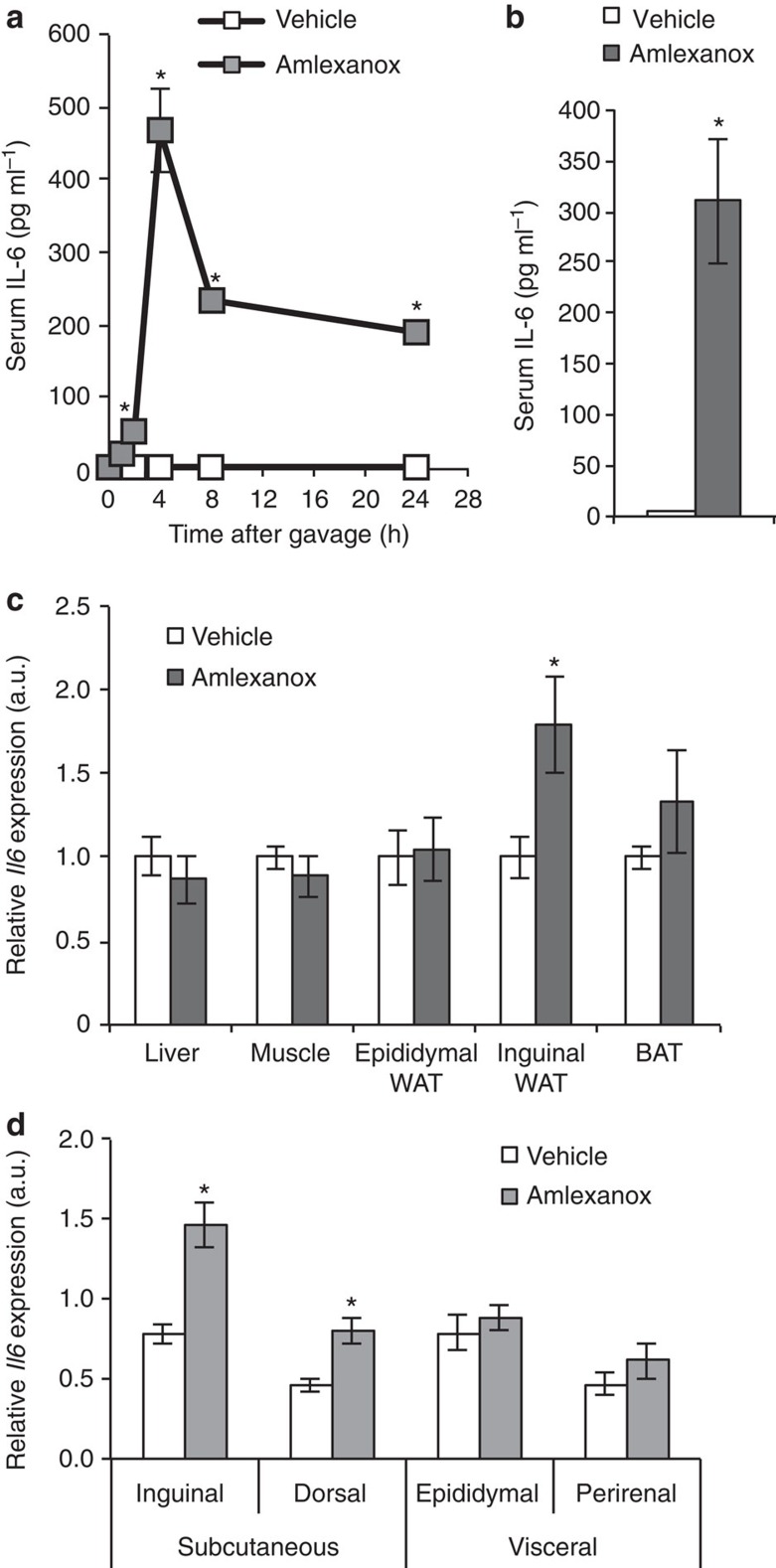
Inhibition of IKK-ε/TBK1 by amlexanox results in IL-6 secretion from subcutaneous adipose tissue. (**a**) Time course of IL-6 secretion after amlexanox administration (*n*=4 mice per time point). (**b**) Serum IL-6 levels 4 h after amlexanox treatment (*n*=8 mice per treatment). (**c**) *Il6* gene expression across tissues. BAT, brown adipose tissue (*n*=8 mice per treatment); WAT, white adipose tissue. (**d**) Relative *Il6* expression in different fat pads (*n*=8 mice per treatment). * indicates a *P* value<0.05 (heteroscedastic *t*-test (**a**,**b**), students *t*-test (**c**,**d**)) amlexanox versus vehicle. Error bars presented as s.e.m.

The preponderance of Jak/Stat responsive genes in the RNA sequencing analysis led us to hypothesize that early effects of amlexanox in the liver might, at least partially, be due to the increased appearance of cytokines in the circulation. We thus screened serum from amlexanox-treated obese mice for a series of cytokines known to be upstream of the Jak/Stat pathway, and identified one, IL-6. Time course studies revealed that serum IL-6 levels were significantly elevated soon after amlexanox treatment, peaking 4 h after gavage with the drug (Fig. 1a,b). Increased serum levels of leukemia-inducible factor were also observed with amlexanox treatment; this IL-6 family ligand may also contribute to the effects of amlexanox on Jak/Stat signalling (Supplementary Fig. 1). No changes in other IL-6 family ligands, including leptin, were observed. To identify the source of IL-6, we performed gene expression analysis in various tissues from mice treated for 4 h with amlexanox. No difference was observed in muscle, liver or epididymal fat *Il6* messenger RNA (mRNA) levels (Fig. 1c). However, there was a significant increase in *Il6* expression in the inguinal adipose tissue. Consistent with preferential induction of *Il6* expression in subcutaneous fat, we also observed increased expression in the dorsal subcutaneous depot, but not in the perirenal visceral fat (Fig. 1d). The precise mechanism regulating the depot specificity of *Il6* expression is unclear, as similar differences are not observed when the tissues are treated *ex vivo*^8^.

### Amlexanox induces *Il6* expression specifically in adipocytes

**Figure 2:**
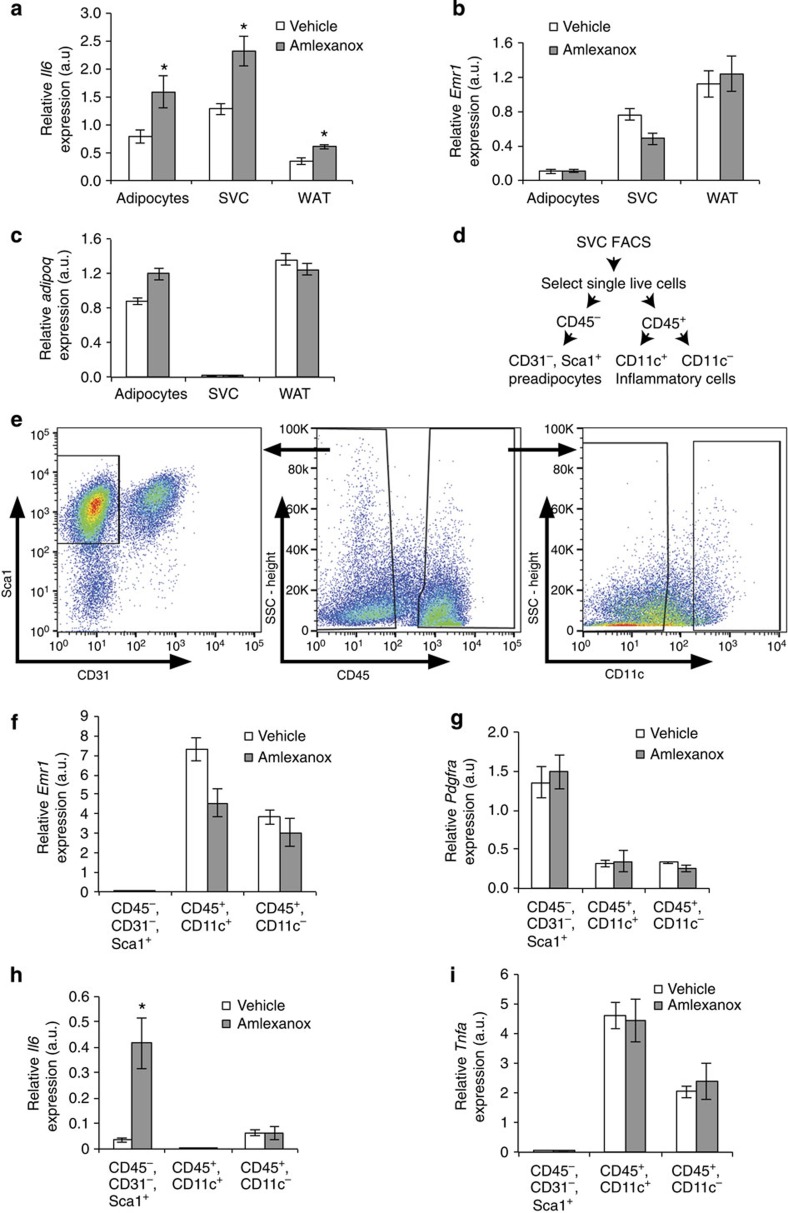
Cellular specificity of amlexanox induced *Il6* expression within fat. (**a**) *Il6* expression in mature adipocytes, SVC and whole inguinal adipose tissue (*n*=8 mice fractionated individually per treatment). (**b**) Relative *Emr1* (F4/80, a macrophage marker) and (**c**) *Adipoq* (a mature adipocyte marker) expression in the different inguinal adipose tissue fractions (*n*=8 mice per treatment). (**d**,**e**) Gating strategy for fluorescence-activated cell sorting (FACS) of SVC. (**f**) Relative *Emr1* and (**g**) *Pdgfra* (a preadipocyte marker) expression in different inguinal stromal cell fractions (*n*=4 groups of 2). (**h**) *Il6* and (**i**) *Tnfa* expression in preadipocytes (CD45^−^, CD31^−^, Sca1^+^) and immune cells (CD45^+^ cells, both CD11c^+^ and CD11c^−^ populations) (*n*=4 separations per treatment each consisting of pooled inguinal fat from 2 mice). * indicates a *P* value<0.05 (heteroscedastic *t*-test) amlexanox versus vehicle. Error bars presented as s.e.m.

To determine which cell type within the subcutaneous fat was responsible for amlexanox-stimulated IL-6 secretion, we fractionated the tissue and measured *Il6* expression levels in the various cell types. Increased *Il6* expression was observed both in mature adipocytes and the stromal vascular cells (SVC) (Fig. 2a). *Emr1* expression was restricted to the SVC, indicating that there was negligible macrophage contamination of the mature adipocyte fraction from the inguinal white adipose tissue (Fig. 2b). As expected, *Adipoq* expression was exclusive to the mature adipocytes (Fig. 2c). We further sorted the SVC to evaluate the amlexanox-induced *Il6* expression in preadipocytes versus immune cells (Fig. 2d,e). *Emr1* expression was exclusive to the CD45^+^ cells, confirming the sorting of macrophages into this population (Fig. 2f). In addition, enriched expression of *Pdgfra* confirmed the identity of the preadipocyte population (Fig. 2g). Amlexanox induced *Il6* expression specifically in the preadipocyte population but not in CD45^+^ cells (Fig. 2h). Importantly other cytokines such as *Tnfa* were specifically expressed in the CD45^+^ immune cells (Fig. 2i). These data indicate that amlexanox specifically directs *Il6* expression in adipocytes as well as preadipocytes, but interestingly not in immune cells such as macrophages.

### Induction of *Il6* expression in adipocytes via cAMP signalling

**Figure 3:**
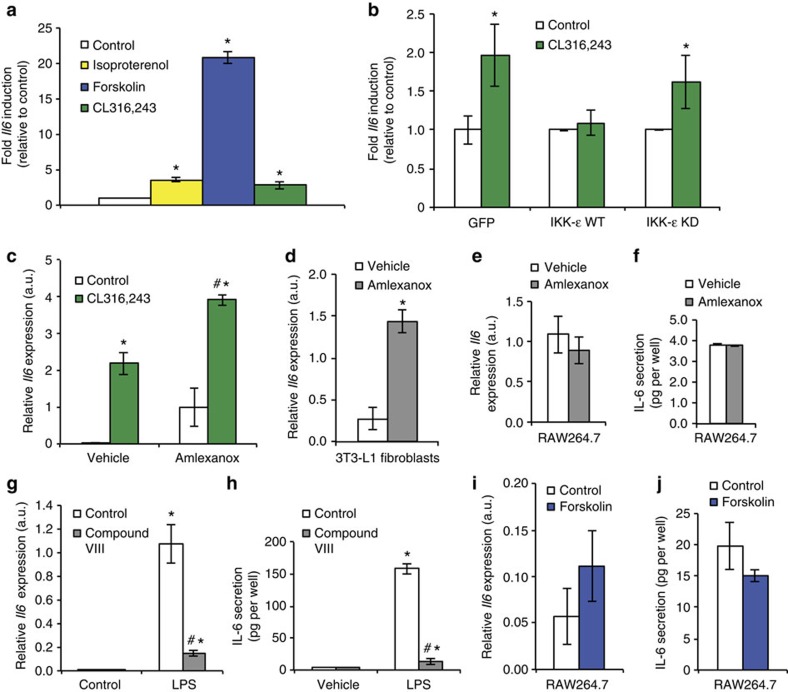
Induction of *Il6* by β-adrenergic signalling in adipocytes. (**a**) Relative induction of *Il6* expression after pharmaceutical activation of β-adrenergic signalling (10 μM isoproterenol and CL316,243) or adenylyl cyclase (50 μM forskolin). (**b**) Relative induction of *Il6* by 10 μM CL316,243 after overexpression of green fluorescent protein control, IKK-ε wild type (WT) or IKK-ε kinase dead mutant (KD). (**c**) Expression of *Il6* in cells treated with 10 μM CL316,243 after 1 h pretreatment with 100 μM amlexanox or vehicle control. (**d**) Relative *Il6* expression in 3T3-L1 fibroblasts after 2 h amlexanox treatment. (**e**) Relative *Il6* expression and (**f**) IL-6 secretion in RAW264.7 cells after 2 h of amlexanox treatment. (**g**) Relative *Il6* expression and (**h**) IL-6 secretion in RAW264.7 cells after 2 h of 0.5 μg ml^−1^ lipopolysaccharide (LPS) treatment, with 1 h pretreatment with 2 μM Compound VIII. (**i**) Relative *Il6* expression and (**j**) IL-6 secretion in RAW264.7 cells after 2 h of 50 μM forskolin treatment. * indicates *P* value<0.05 (student’s *t*-test (**a**,**b**,**d**) or two-way ANOVA plus Tukey–Krammer *post-hoc* analysis; **c**,**g**,**h**) versus control. # indicates *P* value<0.05 (two-way ANOVA plus Tukey–Krammer *post-hoc* analysis) vehicle versus anmelxanox (**c**) or Compound VIII versus control (**g**,**h**). Error bars presented as s.e.m.

In adipocytes, IKK-ε and TBK1 phosphorylate and activate PDE-3B, thereby reducing intracellular cAMP levels and contributing to catecholamine resistance^8^, which has been well-described in obese rodents and patients^9^,^10^,^12^. Inhibition of IKK-ε/TBK1 by amlexanox *in vivo* and *in vitro* relieves this inhibition and restores catecholamine sensitivity^8^. Downstream of cAMP, p38 activation leads to increased expression of *Ucp1* (refs 14, 15, 16), which likely contributes to increased energy expenditure in amlexanox-treated mice. Interestingly, the *Il6* gene is also reported to be a downstream target of cAMP signalling^17^,^18^. Consistently, treatment of 3T3-L1 adipocytes with the β-adrenergic agonists isoproterenol and CL316,243, as well as the adenylyl cyclase activator, forskolin, significantly increased *Il6* expression (Fig. 3a).

To recapitulate the obese state, we overexpressed wild-type IKK-ε in 3T3-L1 adipocytes, which was sufficient to cause catecholamine resistance and block the upregulation of *Il6* in response to CL316,243 (Fig. 3b). Importantly, the kinase dead mutant of IKK-ε was without effect. Furthermore, consistent with an inhibitory effect of IKK-ε and TBK1 on cAMP signalling, pretreatment of cells with amlexanox significantly potentiated the effect of CL316,243 on *Il6* expression (Fig. 3c). Amlexanox also increased *Il6* expression in undifferentiated 3T3-L1 fibroblasts (Fig. 3d), but failed to induce *Il6* expression in the RAW264.7 macrophage cell line (Fig. 3e,f), consistent with the expression pattern described above for SVC *in vivo*. As a positive control, we further investigated *Il6* expression in RAW264.7 macrophages. As expected RAW264.7 cells robustly produce IL-6 in response to lipopolysaccharide, via the NF-κB pathway (Fig. 3g,h). However, unlike adipocytes, RAW264.7 macrophages do not produce IL-6 in response to forskolin, indicating that IL-6 production in macrophages occurs through a pathway different from that used in adipocytes (Fig. 3i,j).

### Amlexanox-induced IL-6 expression is cAMP/p38 dependent

**Figure 4:**
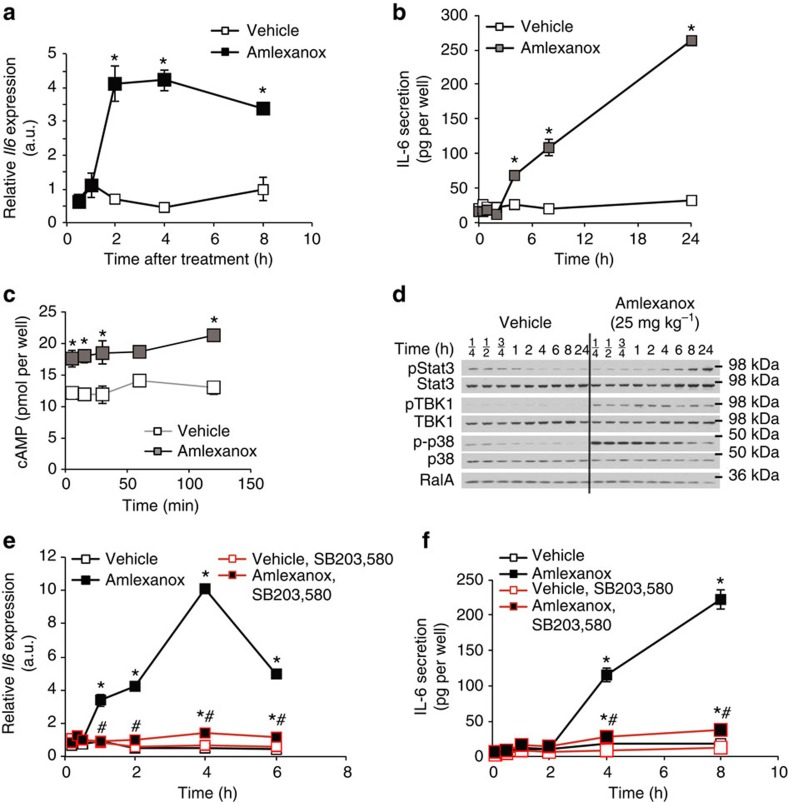
IL-6 secretion from adipocytes by a cAMP/p38-dependent pathway *in vitro*. (**a**) *Il6* expression in 3T3-L1 adipocytes after amlexanox treatment (*n*=3 wells per treatment per time point). (**b**) IL-6 secretion from 3T3-L1 adipocytes after amlexanox treatment (*n*=3 wells per treatment per time point). (**c**) cAMP in 3T3-L1 adipocytes treated with amlexanox for 5, 15, 30, 60 and 120 min (*n*=3 wells per treatment per time point). (**d**) Immunoblot analysis of Stat3, TBK1 and p38 phosphorylation in 3T3-L1 adipocytes treated with amlexanox for indicated times. RalA serves as loading control. (**e**) *Il6* expression in 3T3-L1 adipocytes after amlexanox treatment with and without pre-treatment with SB203,580 (*n*=3 wells per treatment per time point). (**f**) IL-6 secretion from 3T3-L1 adipocytes after amlexanox treatment with and without pre-treatment with SB203,580 (*n*=3 wells per treatment per time point). * indicates *P* value<0.05 (student’s *t*-test **a**–**c** or two-way ANOVA plus Tukey–Krammer *post-hoc* analysis **e**,**f**) amlexanox treated versus vehicle control. # indicates *P* value<0.05 (two-way ANOVA plus Tukey–Krammer *post-hoc* analysis) amlexanox, SB203,580 treated versus amlexanox only. Error bars presented as s.e.m.

Treatment of 3T3-L1 adipocytes with amlexanox alone for 2 h significantly increased *Il6* expression (Fig. 4a). The amlexanox-induced increase in *Il6* expression correlated with secretion of IL-6 from 3T3-L1 adipocytes into the cell culture media 4 h after treatment (Fig. 4b). As predicted by the proposed mechanism involving reduced PDE-3B activity, amlexanox induced a significant increase in cellular cAMP levels in adipocytes (Fig. 4c), confirming that the drug increases IL-6 secretion from adipocytes via increased cAMP signalling.

**Figure 5:**
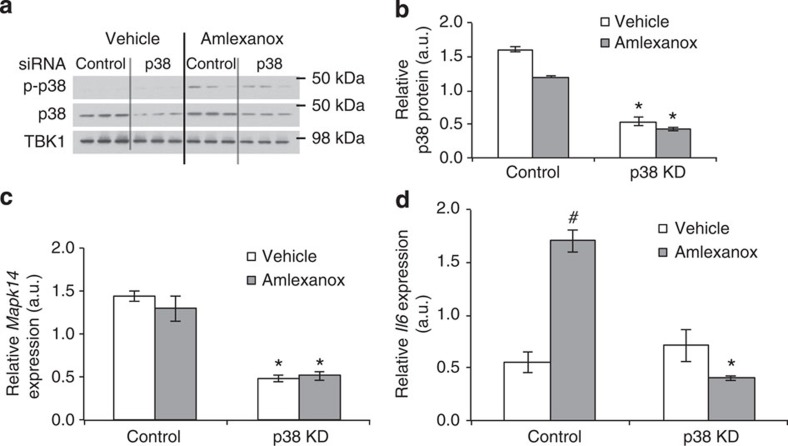
Knockdown of p38 blocks amlexanox-stimulated *Il6* expression in adipocytes. Validated stealth RNAi to *Mapk14* (1320001) and control (12935300) from Life Technologies was electroporated into 3T3-L1 adipocytes on day 1 of 10% FBS only media, and then 4 days after electroporation, vehicle and amlexanox treatments were administered after a 3-h serum starve. (**a**) Immunoblots for total and phosphorylated p38 protein. (**b**) quantification of immunoblots in **a**. (**c**) Further validation of p38 knockdown using Q-PCR to measure *Mapk14* mRNA levels. (**d**) mRNA levels of *Il6* after 4 h of amlexanox treatment in control and p38 knockdown cells (*n*=3 wells per condition). * indicates *P* value<0.05 p38 KD versus control, # indicates *P* value<0.05 amlexanox versus vehicle (two-way ANOVA plus Tukey–Krammer *post-hoc* analysis). Error bars represent s.e.m.

Induction of *Il6* by cAMP has been previously shown to be dependent on p38 (refs 18, 19, 20). Consistent with this notion, p38 phosphorylation increased after amlexanox treatment (Fig. 4d and Supplementary Fig. 2). Notably, the stimulation of p38 phosphorylation by amlexanox was rapid, occurring over a time course similar to its ability to relieve the feedback inhibition of TBK1 phosphorylation^8^. The induction of Stat3 phosphorylation in these adipocytes was not observed until after 4 h of treatment, a lag consistent with the time course of secretion of IL-6 into the media. Further supporting that *Il6* induction by amlexanox is mediated by a p38-dependent pathway downstream of cAMP, pretreatment of adipocytes with the p38 inhibitor SB203,580 completely blocked the effect of amlexanox to increase both *Il6* expression and IL-6 secretion (Fig. 4e,f). The dependency of amlexanox-induced IL-6 secretion in adipocytes on p38 was confirmed by short interfering RNA-mediated knockdown of p38 (Mapk14) prior to amlexanox treatment (Fig. 5 and Supplementary Fig. 3). To verify that these effects of amlexanox were mediated by its inhibition of IKK-ε/TBK1, we utilized another structurally unrelated inhibitor of these kinases CAY-10576. Treatment of 3T3-L1 adipocytes with CAY-10576 also increased the expression and secretion of IL-6 in a p38-dependent manner (Supplementary Fig. 4), confirming that this effect is due to inhibition of IKK-ε/TBK1, and not a non-specific effect of amlexanox.

### Amlexanox-induced IL-6 signalling is p38-dependent *in vivo*

**Figure 6:**
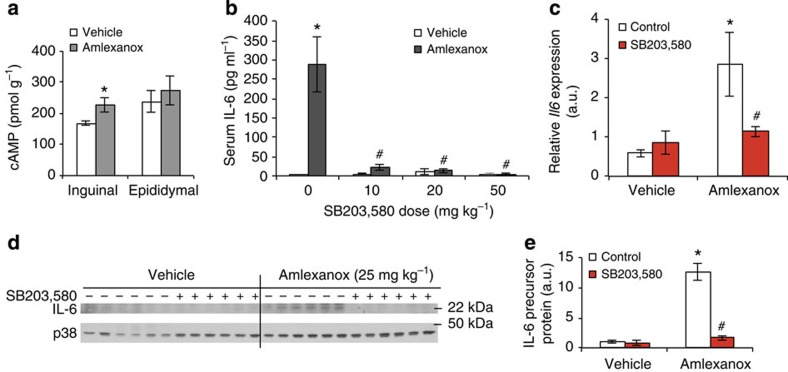
IL-6 secretion from adipocytes by a cAMP/p38-dependent pathway *in vivo*. (**a**) cAMP levels in adipose tissue 4 h after amlexanox treatment *in vivo* (*n*=8 mice per treatment). (**b**) Serum IL-6 levels 4 h after amlexanox treatment following SB203,580 pre-treatment (*n*=6 amlexanox-treated and 5 vehicle control mice per SB203,580 dose). (**c**) *Il6* expression and (**d**) IL-6 precursor protein levels in inguinal adipose tissue 4 h after amlexanox treatment following SB203,580 pre-treatment, quantification in left panel (*n*=6 mice per treatment). (**e**) quantification of immunoblots in **d**. * indicates *P* value<0.05 (student’s *t*-test **a** or two-way ANOVA plus Tukey–Krammer *post-hoc* analysis **b**–**e**) amlexanox treated versus vehicle control. # indicates *P* value<0.05 (two-way ANOVA plus Tukey–Krammer *post-hoc* analysis) amlexanox, SB203,580 treated versus amlexanox only. Error bars represent s.e.m.

We also explored the role of the cAMP/p38 pathway *in vivo*. Similar to the induction of *Il6* expression, amlexanox treatment specifically increased cAMP levels in inguinal adipose tissue (Fig. 6a). To demonstrate that the stimulatory effect of amlexanox on IL-6 secretion was downstream of this pathway, we pretreated mice by oral gavage with SB203,580. The stimulatory effect of amlexanox on serum IL-6 levels was inhibited by p38 blockade (Fig. 6b). Consistent with p38-dependent induction of IL-6 in inguinal adipose tissue by amlexanox, we observed a significant increase in *Il6* mRNA levels as well as IL-6 precursor protein levels, both of which were completely blocked by administration of SB203,580 (Fig. 6c–e and Supplementary Fig. 5).

### Hepatic Stat3 activation is observed with amlexanox treatment

**Figure 7:**
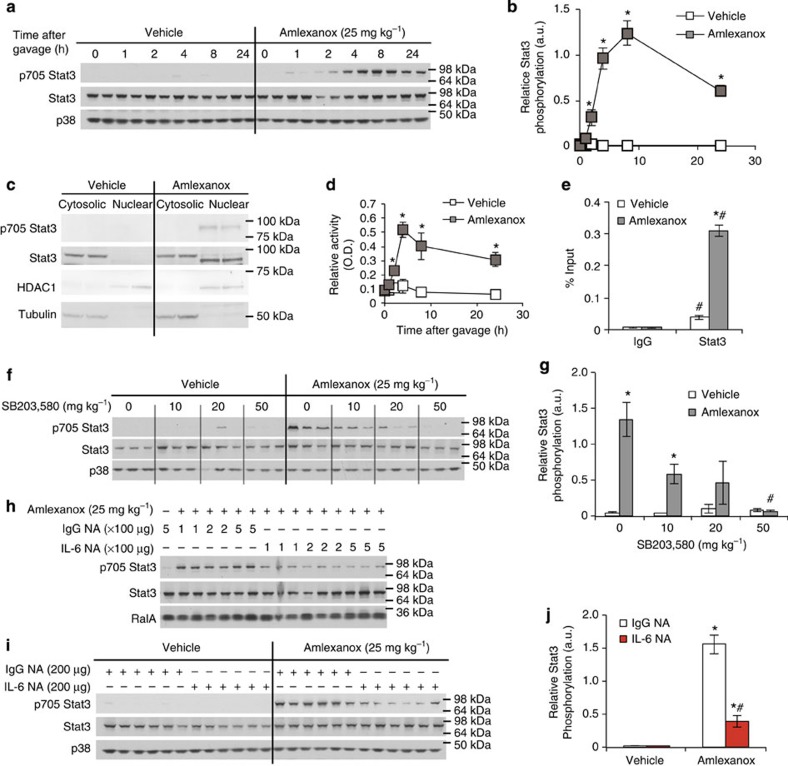
IL-6 induced hepatic Stat3 activation. (**a**) Time course of the effects of amlexanox treatment on hepatic Stat3 phosphorylation. (**b**) quantification of immunoblots in **a**. (**c**) Immunoblot analysis of STAT3 and phosphorylated STAT3 in nuclear and cytosolic fractions after 8 h treatment with amlexanox or vehicle control. HDAC1 and Tubulin are positive controls for the nuclear and cytosolic fractions, respectively. (**d**) Stat3 and IgG control ChIP at Stat3 binding site in the *Socs3* promoter (*n*=3). (**e**) Stat3 DNA binding activity after amlexanox treatment *in vivo* (*n*=4 mice per time point). (**f**) Hepatic Stat3 phosphorylation in amlexanox-treated and vehicle control mice with varying doses of pretreatment with SB203,580. (**g**) quantification of immunoblots in **f**. (**h**,**i**) Hepatic Stat3 phosphorylation in amlexanox-treated and vehicle control mice after pretreatment with either IL-6 neutralizing antibody or sera-type matched IgG control antibody; (**h**) 4 h after amlexanox gavage and (**i**) 8 h after amlexanox gavage. (**j**) quantification of immunoblots in **i**. * indicates *P* value<0.05 (student’s *t*-test **b**,**e** and two-way ANOVA plus Tukey–Krammer *post-hoc* analysis **d**,**g**,**j**) amlexanox treated versus vehicle control. # indicates *P* value<0.05 (ANOVA plus Tukey–Krammer *post-hoc* analysis) Stat3 versus IgG in **d**, amlexanox, SB203,580 treated versus amlexanox only in **g** and IL-6 NA versus IgG NA in **f**. Error bars represent s.e.m.

Next, we investigated whether the increase in serum IL-6 after amlexanox treatment was associated with increased Stat3 activity in the liver. Following administration of amlexanox to obese mice, we assayed phosphorylation of Stat3 at tyrosine 705, which mediates dimerization and subsequent nuclear localization and transcriptional regulation. Hepatic Stat3 phosphorylation was increased 4 h after amlexanox treatment, coincident with the increase in serum IL-6 levels (Figs 7a,b and 1a, respectively, Supplementary Fig. 6). As expected, Stat3 phosphorylation was specifically associated with nuclear localization and DNA binding in livers from the amlexanox-treated mice (Fig. 7c,d, respectively, Supplementary Fig. 7). Furthermore, amlexanox treatment significantly enriched Stat3 occupancy at the promoter of *Socs3*, a well-known Stat3 target gene (Fig. 7e).

### Activation of hepatic Stat3 is dependent on IL-6 signalling

Pre-treatment with SB203,580 dose dependently decreased amlexanox-stimulated hepatic Stat3 phosphorylation, coordinating well with decreases in serum IL-6 levels (Figs 7f,g and 6b, respectively, Supplementary Fig. 8). To causally link serum IL-6 to hepatic Stat3 phosphorylation, we utilized an IL-6 neutralizing antibody. We tested a range of neutralizing antibody doses, all of which efficiently blocked amlexanox-stimulated hepatic Stat3 phosphorylation (Fig. 7h and Supplementary Fig. 9). While the IgG control antibody had no effect on the amlexanox or vehicle control-treated mice, the IL-6 neutralizing antibody blocked the stimulation of Stat3 phosphorylation in the amlexanox-treated mice, but had no effect in the vehicle-treated mice (Fig. 7i,j and Supplementary Fig. 10). We opted to use neutralizing antibodies instead of genetic knockout of *Il6* in these acute studies, thus avoiding confounding effects of IL-6 deficiency throughout development and on responses to diet.

### Suppression of *G6pc* expression by Stat3

**Figure 8:**
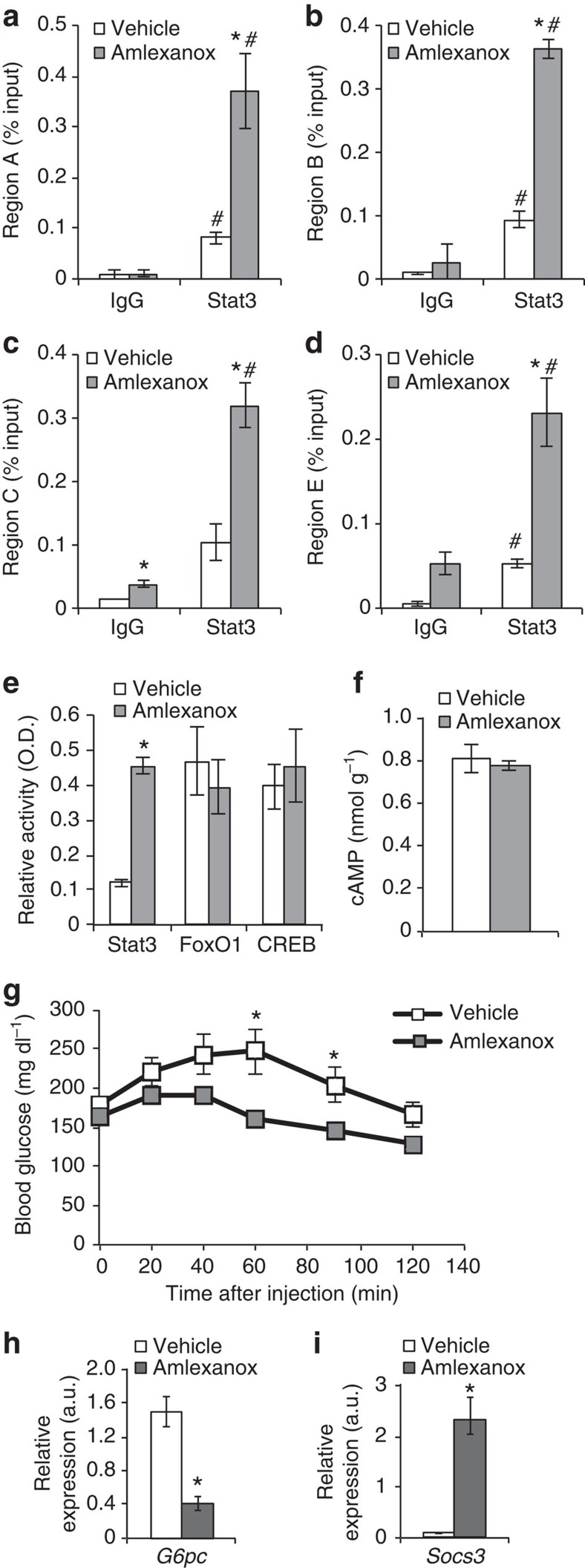
Amlexanox reduces hepatic glucose production via suppression of G6pc. (**a**–**d**) Stat3 and IgG control ChIP of regions A–C and E of the *G6pc* promoter (*n*=3). (**e**) DNA binding activity of transcription factors known to regulate *G6pc* expression (liver nuclear lysates from eight mice per condition). (**f**) Liver cAMP levels 4 h after amlexanox treatment (*n*=8 mice per treatment). (**g**) Pyruvate tolerance test in amlexanox and vehicle control-treated mice (*n*=6 mice per treatment). Hepatic expression of (**h**) *G6pc* and (**i**) *Socs3* in amlexanox and vehicle control-treated mice, respectively, (*n*=8 mice per treatment). * indicates *P* value<0.05 amlexanox treated versus vehicle control. # indicates *P* value<0.05 Stat3 versus IgG (two-way ANOVA plus Tukey–Krammer *post-hoc* analysis **a**–**d** and student’s *t*-test **e**–**i**). Error bars represent s.e.m.

**Figure 9:**
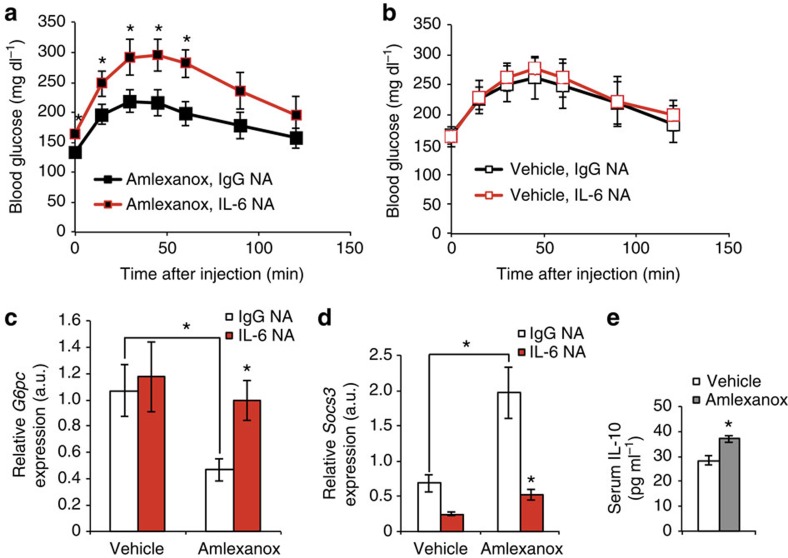
Amlexanox reduces hepatic glucose production in an IL-6-dependent manner. Pyruvate tolerance test in mice pretreated with either IL-6 neutralizing antibody (NA) or serotype-matched IgG control antibody (*n*=6 mice per treatment) before either (**a**) amlexanox or (**b**) vehicle control treatment. Hepatic expression of (**c**) *G6pc* and (**d**) *Socs3* in amlexanox-treated and vehicle control mice after pretreatment with either IL-6 NA or sera-type matched IgG control antibody (*n*=6 mice per treatment). (**h**) Serum IL-10 levels 4 h after amlexanox or vehicle control treatment. * indicates *P* value<0.05 (student’s *t*-test **a**,**b**,**e** and ANOVA plus Tukey–Krammer *post-hoc* analysis **c**,**d**) IL-6 neutralizing antibody versus IgG control and amlexanox treated versus vehicle control (**e** and as indicated in **c**,**d**) or error bars represent s.e.m.

In the liver, Stat3 activity is associated with suppression of gluconeogenic gene expression, specifically *G6pc* (refs 21, 22, 23). Increased binding of Stat3 to the *G6pc* promoter at all four previously characterized binding regions was observed in liver derived from amlexanox-treated mice (Fig. 8a–d). Amlexanox treatment specifically increased DNA binding by Stat3, but not FoxO1 or CREB, indicating that the latter transcription factors are likely not involved in the regulation of *G6pc* expression by the drug (Fig. 8e). Consistent with the indirect effects of amlexanox in the liver and lack of effect on CREB, amlexanox did not increase cAMP levels in liver (Fig. 8f). We verified that treatment with amlexanox results in reduced gluconeogenesis in obese mice after only 3 days of treatment, by performing a pyruvate tolerance test (Fig. 8g). Consistent with Stat3 activation by IL-6, the reduction in hepatic glucose production was associated with reduced expression of *G6pc*, and increased expression of *Socs3* (Fig. 8h,i). Furthermore, treatment with an IL-6 neutralizing antibody completely blocked the anti-gluconeogenic effects of amlexanox treatment, as determined by a pyruvate tolerance test (Fig. 9a,b). The IL-6 neutralizing antibody also blocked the suppression of gluconeogenic gene expression by amlexanox, as well as the induction of *Socs3* expression (Fig. 9c,d).

### Long-term benefits could be mediated by reduced inflammation

The exact role of IL-6-mediated suppression of gluconeogenesis in the long-term insulin-sensitizing effects of amlexanox remains to be fully elucidated. IL-6 signalling has previously been shown to have anti-inflammatory effects in liver as well as inflammatory cells^24^,^25^. Therefore, it is possible that IL-6 stimulates the resolution of chronic low-grade inflammation, as seen with long-term amlexanox treatment^13^. Interestingly, serum IL-10 levels increase after both short and long-term amlexanox treatment (Fig. 9e)^13^.

### Potential relevance to human disease

**Figure 10:**
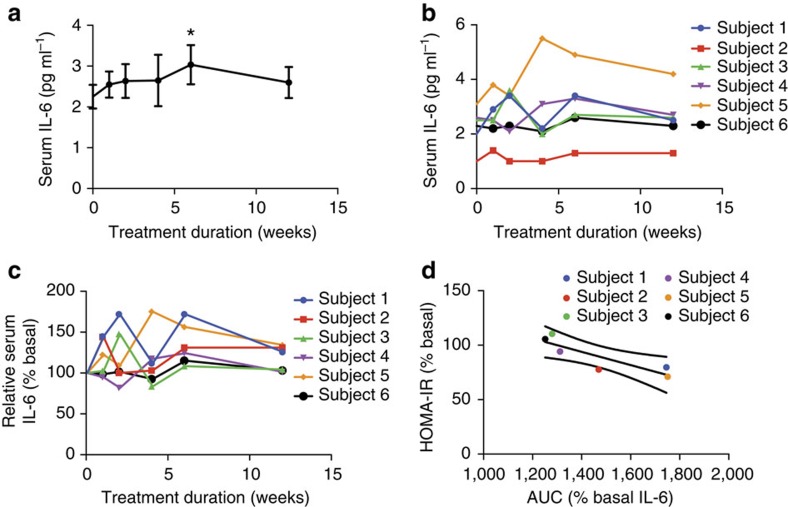
Improved HOMA-IR correlates with IL-6 response in human subjects treated with amlexanox. (**a**) Average serum IL-6 levels in human subjects over the course of 12 weeks of amlexanox treatment (*n*=6). * indicates *P* value<0.05 (paired *t*-test) indicated time point versus baseline. Error bars represent s.e.m. (**b**) Individual serum IL-6 levels in human subjects over the course of 12 weeks of amlexanox treatment (*n*=6). (**c**) Individual baseline normalized IL-6 levels over the course of 12 weeks of amlexanox treatment (*n*=6). (**d**) Linear regression of change in HOMA-IR score with baseline normalized IL-6 area under curve (AUC). Y-axis: % basal HOMA-IR=(HOMA-IR after 12 weeks of treatment)/(HOMA-IR at baseline) × 100%. X-axis: AUC (% basal serum IL-6 over the 12 weeks of treatment—such that a value of 1,200 indicates no response and a value of 1,800 corresponds with an average 1.5-fold increase in serum IL-6 levels over the 12 weeks of treatment).

To explore whether a similar crosstalk occurs in humans, we investigated the relationship between circulating IL-6 levels and measures of insulin sensitivity in a small cohort of obese-diabetic patients treated with amlexanox in a 12-week open-label clinical trial. In the mice, we observed the most robust effects on IL-6 signalling over the first 24 h of drug treatment. However, the earliest serum withdrawal in this clinical trial was 1 week after initiation of treatment. In addition, differences in the dosage of amlexanox in this clinical trial further obfuscate direct comparison with the IL-6 response in the animal studies. Nevertheless, a relatively small but statistically significant increase in serum IL-6 levels was observed after the sixth week of treatment, in these patients (Fig. 10a). Increased serum IL-6 levels were also observed during the first 2 weeks of treatment in four of the six patients (Fig. 10b). The relative increase in serum IL-6 in response to amlexanox treatment varied from patient to patient, with some patients showing a more pronounced response than others, even after baseline normalization (Fig. 10c).

Although fasting blood glucose levels and the homeostasis model assessment-estimated insulin resistance (HOMA-IR) score tended to be lower after amlexanox treatment, these results did not reach statistical significance in this small cohort (Supplementary Fig. 11). Hypothesizing that the insulin-sensitizing effect of amlexanox treatment in humans is associated with the relative IL-6 response, we performed a linear regression analysis to determine whether the IL-6 response correlated with improvements in the HOMA-IR score, as a measure of insulin sensitivity^26^,^27^,^28^. To quantify the IL-6 responsiveness for each patient, we calculated the area under the curve for serum IL-6 levels after normalization to baseline for each patient. We calculated the HOMA-IR score at the end of the 12-week study as a per cent of baseline HOMA-IR for each individual patient. There was a significant correlation (*P* value=0.02, *R*^2^ value=0.8) between the IL-6 response and improvements in the HOMA-IR score (Fig. 10d). Although we have limited power in a cohort of this size, these data are consistent with the subcutaneous adipose tissue–liver communication axis that we observed in mice treated with amlexanox. The complete results of this clinical study will be reported elsewhere.

## Discussion

The role of IL-6 in regulation of glucose homoeostasis has been controversial^29^. IL-6 levels are chronically increased in obesity and correlate with insulin resistance^30^,^31^,^32^,^33^, and reports suggest that the cytokine might contribute to insulin resistance through induction of *Socs3* in hepatocytes^34^,^35^. However, other studies have suggested a beneficial effect of the cytokine^36^,^37^,^38^. Differences in the chronic versus acute metabolic effects of IL-6 may explain why contradictory results have been observed in genetic mouse models of disrupted IL-6 signalling. It is acutely secreted from skeletal muscle in response to exercise, and studies have indicated that it is produced from brown or white fat depots in response to sympathetic activation^17^,^18^,^39^,^40^,^41^,^42^. Adding further complexity to the metabolic effects of IL-6, our results indicate that IL-6 may also represent a crucial player in a subcutaneous adipose tissue–liver axis.

Taken together, these data indicate a new, previously unappreciated crosstalk between subcutaneous adipose tissue and liver in the control of glucose homoeostasis. These findings suggest that activation of the inflammatory programme in obesity induces expression of IKK-ε/TBK1 in both adipocytes and preadipocytes of subcutaneous fat, initiating a process of catecholamine resistance that reduces energy expenditure, but also suppresses the catecholamine-induced increase in adipose IL-6 expression, thereby contributing to elevated hepatic glucose production in obesity. Acute inhibition of IKK-ε/TBK1 by amlexanox reverses this catecholamine resistance, raising cAMP levels in subcutaneous adipose tissue, and in the process dramatically increasing IL-6 expression and subsequent activation of Stat3 in the liver to suppress gluconeogenesis. These studies reinforce the notion of IKK-ε/TBK1 inhibition as a promising therapeutic strategy for the treatment of insulin resistance in obese patients.

## Methods

### Reagents

All chemicals were obtained from Sigma-Aldrich unless otherwise stated. TBK1 (3013), phosphorylated TBK1 (Ser172-5483) diluted 1:500, Stat3 (9139), phosphorylated Stat3 (Tyr705-9131), p38 (9212), phosphorylated p38 (Thr180/Tyr182-9211), tubulin (2128)-specific antibodies were purchased from Cell Signaling. HDAC1 (H-51) and Stat3 (C-20)-specific antibodies were purchased from Santa Cruz. RalA (610222)-specific antibody was obtained from BD Bioscience and used at a dilution of 1:3,000. IL-6 antibody was obtained from Abcam (ab6672). All antibodies were diluted 1:1,000 unless otherwise noted. EDTA-free protease inhibitor tablet was purchased from Roche Diagnostics. For *in vitro* experiments InSolution SB203,580 (1 mg ml^−1^) was purchased from Calbiochem-EMD. For *in vivo* experiments SB203,580 powder was purchased from Selleck Chemicals.

### Animals

We fed wild-type male C57Bl/6 mice a high fat diet consisting of 45% of calories from fat (D12451 Research Diets Inc.) starting at 8 weeks of age. Amlexanox was administered by daily oral gavage at a dose of 25 mg kg^−1^. SB203,580 was administered by oral gavage at the indicated doses 30–60 min prior to amlexanox administration. Mice were preconditioned to the oral gavage for a minimum of 1-week daily gavage with an equivalent volume of saline. Neutralizing antibodies (R&D AB-406-NA and AB-108-C) were administered by intraperitoneal injection just prior to oral gavage. During metabolic studies, such as pyruvate tolerance tests, ear tag numbers were used to identify individual animals and the researcher performing the test was blinded to the treatment group assignment during data collection. Cohorts of 20–44 mice were produced in up to 20 in-house breeding cages, and constructed to minimize the birthdate range. Mice were assigned to treatment groups, such that prior to treatment the mean body weight, as well as the s.d. of the body weight, was equal across treatment groups, using a method similar to block randomization. Additional consideration was given to housing, such that each cage contained multiple treatment groups, to avoid confounding by cage effects. Sample size was determined based on the available cohorts and the number of treatment groups required. Mice were housed in a specific pathogen-free facility with a 12-h light, 12-h dark cycle and given free access to food and water, except when food was restricted during fasting. All animal use was in compliance with the Institute of Laboratory Animal Research Guide for the Care and Use of Laboratory Animals and approved by the University Committee on Use and Care of Animals at the University of Michigan.

### Pyruvate tolerance test

Fasting blood glucose levels from tail blood were measured after a 16-h fast, using the OneTouch Ultra glucometer (Lifescan). Mice were then given an intraperitoneal injection of sodium pyruvate (P2256) at a dose of 1.5 g kg^−1^ body weight. Blood glucose levels were measured again at 15, 30, 45, 60, 90 and 120 min post injection.

### 3T3-L1 differentiation

3T3-L1 fibroblasts (American Type Culture Collection) were cultured in DMEM containing 10% FBS. Once grown to confluence, adipocyte differentiation was initiated using a three component cocktail containing 500 μM 3-Isobutyl-1-methylxanthine, 250 nM dexamethasone and 1 μg ml^−1^ insulin for the first 3 days, followed by an additional 3 days of media containing insulin and finally differentiation was completed in the culture media. Only cultures in which >90% of cells displayed adipocyte morphology were used. Fully differentiated adipocytes, which had been cultured in the FBS only media for 1 week, were serum starved for 3 h in media containing 0.5% FBS, then treated with 100 μM amlexanox or vehicle control. Where applicable, cells were pretreated for 30 min with 5 mM SB203,580 or vehicle control.

### IL-6 measurement

Mouse IL-6 levels were quantified using Mouse IL-6 Quantikine ELISA from R&D (SM6000B), with 50 μl of serum or cell culture media. Human IL-6 serum levels were quantified using the Human IL-6 Quantikine ELISA Kit (D6050) with 100 μl serum.

### cAMP measurement in cells and tissues

Tissue cAMP levels were determined using the cAMP enzyme immunoassay kit (Sigma CA201) according to the manufacturer’s instructions. Cellular cAMP levels were determined using the cAMP enzyme immunoassay kit (Sigma CA200) according to the manufacturer’s instructions.

### Western blot analysis

We homogenized tissues in lysis buffer (50 mM Tris pH 7.5, 150 mM NaCl, 2 mM EDTA, 10% glycerol, 1% Triton X-100, 1 mM dithiothreitol, 1 mM Na_3_VO_4_, 5 mM NaF, 1 mM phenylmethanesulfonylfluoride, 25 mM glycerol 2-phosphate and freshly added protease inhibitor tablet) and then incubated them for 1 h at 4 °C. Cell lysates were produced in an SDS lysis buffer (100 mM Tris pH 7.5, 130 mM NaCl, 1% NP-40, 0.1% SDS, 0.2% sodium deoxycholate, 1 mM Na_3_VO_4_, 1 mM NaF, 100 mM Na_4_P_2_O_7_, 1 mM phenylmethanesulfonylfluoride, 25 mM glycerol 2-phosphate and freshly added protease inhibitor tablet) and sonicated for three 5 s pulses at an output power of 6. We centrifuged crude lysates at 14,000 *g* for 15 min twice and determined the protein concentration using Bio-Rad Protein Assay Dye Reagent. Samples were diluted in SDS sample buffer. Bound proteins were resolved by SDS–PAGE and transferred to nitrocellulose membranes (Bio-Rad). Individual proteins were detected with specific antibodies and visualized on film using horseradish peroxidase-conjugated secondary antibodies (Bio-Rad) and Western Lightning Enhanced Chemiluminescence (Perkin Elmer Life Sciences).

### SVC isolation and fluorescence-activated cell sorting

The stromal vascular fraction was separated from adipocytes by centrifugation after digestion. Excised white adipose tissue was digested in RPMI with 0.5% BSA and 1 mg ml^−1^ type II collagenase for 30 min at 37 °C with gentle agitation. Then the cell suspension was filtered through a 100 μm filter and centrifuged at 700 *g* for 5 min to separate floating adipocytes from SVC pellet, and RPMI with 0.5% BSA was used to wash cells. The following antibodies were used for staining: anti-CD45(30-F11), anti-CD31(390), anti-CD11c(N418) and anti-Sca-1(D7), all from eBioscience. Stained SVC were sorted by flow cytometry on a MoFlo Astrios cell sorter (Beckman Coulter) with the help of the University of Michigan’s Flow Cytometry Core.

### Gene expression analysis

RNA extractions from tissues were performed using the RNeasy Lipid Tissue Kit (Qiagen) While RNA extraction from cells utilized the RNeasy Kit, we used the Superscript First-Strand Synthesis System for reverse transcription–PCR (Invitrogen) with a 3:1 mixture of random hexamers/oligo dT primers for reverse transcription. Real-time PCR amplification was performed on samples in triplicate with Power SYBR Green PCR Master Mix (Applied Biosystems) using the Applied Biosystems 7900HT Fast real-time PCR System and quantified using an internal standard curve and *Arbp* as the control gene. The sequences of all primers used in this study are listed in Supplementary Table 2.

### RNA sequencing

Total RNA from mouse livers was isolated using Trizol (Invitrogen) and the RNeasy mini kit according to the manufacturer’s instructions. Biological triplicates of amlexanox-treated and non-treated tissues were used to prepare sequencing libraries from 100–500 ng total RNA using the TruSeq RNA Sample Preparation Kit v2 (Illumina) according to the manufacturer’s protocol.

*Sample preparation*. Briefly, mRNA was purified, fragmented and used for first- and second-strand complementary DNA synthesis followed by adenylation of 3′ ends. Samples were ligated to unique adapters and subjected to PCR amplification. Libraries were then validated using the 2100 BioAnalyzer (Agilent), normalized and pooled for sequencing on the Illumina HiSeq 2000 using bar-coded multiplexing and a 100 bp read length.

*Data analysis*. Read alignment and junction mapping was accomplished using TopHat2 v2.0.4 using a 25 bp 5′ segment seed for initial mapping followed by differential gene expression analysis using Cuffdiff v2.0.2 to map reads to the reference genome annotation, NCBI mouse build 37.2 (ref. 43). Median sequencing read yield per replicate sample was 24.2 M. Data were expressed as fragments per kilobase of exon per million fragments mapped . Volcano plots were generated from Cuffdiff output using CummeRbund v2.0.0 (ref. 43).

*RNA-seq library construction*. Single-cell complementary DNA size distribution and concentration were assessed on a capillary electrophoresis-based fragment analyser (Advanced Analytical). Illumina libraries were constructed in 96-well plates using the Illumina Nextera XT DNA Sample Preparation kit as described previously using the protocol supplied by Fluidigm. For each C1 experiment, a bulk RNA control (about 200 cells) and a no-cell negative control were processed in parallel in PCR tubes, using the same reagent mixes as used on chip. Libraries were quantified by Bioanalyzer, using High Sensitivity DNA analysis kit, and also fluorometrically using Qubit dsDNA HS Assay kits and a Qubit 2.0 Fluorometer (Invitrogen, Life Technologies).

*DNA sequencing*. Single-cell Nextera XT (Illumina) libraries of one experiment were pooled and 100 bp) paired-end were sequenced on Illumina HiSeq 2000 to a depth of (2–6) × 106 reads (three replicate experiments of distal mouse lung epithelial cells at E18.5, one experiment at E14.5 and one experiment on adult AT2 cells) or 150 bp paired-end on Illumina MiSeq (one experiment at E16.5) to a depth of 100,000–550,000 reads with v3 chemistry. CASAVA 1.8.2 was used to separate out the data for each single cell by using unique barcode combinations from the Nextera XT preparation and to generate *.fastq files.

### KEGG pathway analysis

Normalized relative fold expression levels from RNA-Seq were calculated for selected genes and differentially expressed genes were determined. Pathway analysis was performed with DAVID Bioinformatics Resources 6.7 ( http://david.abcc.ncifcrf.gov/)^44^,^45^.

### Chromatin immunoprecipitation

Flash-frozen vehicle or 8 h amlexanox-treated liver samples (~400 mg per chromatin immunoprecipitation (ChIP)) were thawed on ice and homogenized in 10 volumes 2 mM DSG (ProteoChem), incubated for 10 min at room temperature and then passed through a 70 μM mesh to remove extracellular debris. The homogenized cells were centrifuged, and pellets were resuspended in 1% formaldehyde in PBS and incubated at room temperature for 10 min. Formaldehyde crosslinking was quenched by the addition of glycine to a final concentration of 125 mM, and then incubated at room temperature for 5 min. The crosslinked cells were centrifuged and cell pellets were resuspended in ice cold Cell Lysis Buffer to isolate nuclei. The nuclei were collected by centrifugation, and then sonicated and chromatin was immunoprecipitated with 5 μg of Stat3 antibody (C-20) or normal rabbit IgG (Cell Signaling, 2729) overnight. Immunoprecipitated complexes were captured with protein A dynabeads (novex) for 90 min at 4 °C, washed and eluted from beads. Complexes were then decrosslinked, DNA was purified and real-time PCR was performed. Primer sequences used for ChIP can be found in Supplementary Table 3.

### Transcription factor DNA binding assays

Nuclear extracts from liver were prepared using a nuclear extraction kit (Active Motif 40010). Ten nanograms of nuclear extract were used to assay specific transcription factor DNA binding activity using TransAM kits (Active Motif 45196, 46396 and 42092). These kits utilize response element DNA affixed to the plate and specific primary antibodies to quantify the relative DNA binding activity of specific transcription factors.

### Clinical trial

Six obese type-2 diabetic patients (body mass index range 31 to 39 kg m^−2^, two males and four females, ages 24 to 68 years) were enroled in clinical trial NCT01842282. After patients gave informed consent, they were instructed to take amlexanox tablets (Takeda) in an open-label manner at a dosage of 25 mg three times daily. After 2 weeks, the dosage was increased to 50 mg tablets three times daily. Blood collection was performed at baseline and 1, 2, 4, 6 and 12 weeks of treatment, serum samples were frozen at −80 °C for future combined analysis. Patients were provided instructions for a standardized meal (600 kcal, 55% carbohydrates, 30% fat and 15% protein) and then instructed to fast overnight after 2200 hours prior to the baseline and 12-week visits, such that HOMA-IR could be calculated from measurements of fasted blood glucose and serum insulin. The study was reviewed and approved by the University of Michigan Medical School Institutional Review Board (IRBMED; HUM00065177).

### Statistics

Averaged values are presented as the mean±s.e.m. Equality of variance was determined using an *f*-test. Data were determined to be normally distributed as the maximum and minimum values in each data set were <3 s.d. from the mean. When comparing the two groups, statistical significance was determined using the student’s *t*-test; except when the *f*-test indicated statistically different variances (*P* value<0.05), then a heteroscedastic *t*-test was used. When more than two groups were investigated, we first performed an analysis of variance (ANOVA) to establish that not all groups were equal. Following a statistically significant ANOVA, we performed between group comparisons using the Tukey–Krammer *post-hoc* analysis. Linear regression was used to determine correlation significance, and displayed as the best fit line with a 95% confidence band. Linear regression, ANOVA and Tukey–Krammer tests were performed using Prism version 6.0 (ref. 46).

## Additional information

**How to cite this article:** Reilly, S. M. *et al*. A subcutaneous adipose tissue–liver signalling axis controls hepatic gluconeogenesis. *Nat. Commun.* 6:6047 doi: 10.1038/ncomms7047 (2015).

**Accession codes**: The RNA sequencing data have been deposited in NCBI’s Gene Expression Omnibus under accession code GSE57054.

## Supplementary Material

Supplementary Information Supplementary Figures 1-11 and Supplementary Tables 1-3
