# Supplementary material for: A subcutaneous adipose tissue–liver signalling axis controls hepatic gluconeogenesis
Source: Nat Commun. 2015 Jan 12;6:6047. doi: 10.1038/ncomms7047 (PMC4324568; doi:10.1038/ncomms7047)
Supplement: Supplementary Information — Supplementary Figures 1-11 and Supplementary Tables 1-3 [file ncomms7047-s1.pdf]

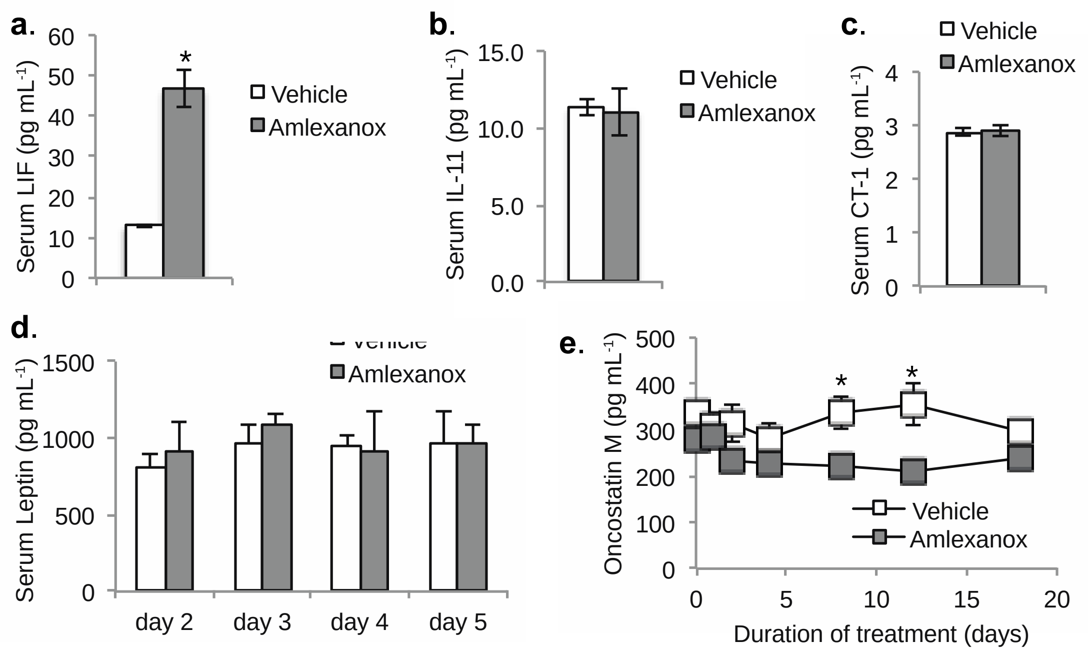

Supplementary Figure 1: **Induction of IL-6 family cytokines by amlexanox**. Serum levels of (**a**) leukemia inducible factor (LIF), (**b**) IL-10 and (**c**) Cardiotrophin-1 (CT-1) four hours after amlexanox treatment. (**d**) Serum leptin levels after amlexanox treatment. (**e**) Serum oncostatin M levels after amlexanox treatment. Differences in oncostatin M disappear when normalized to baseline. Serum cytokine and adipokine levels were quantified using the Mouse LIF Quantikine ELISA Kit (MLF00), Mouse IL-11 DuoSet (DY418), Mouse Cardiotrophin-1 (CT-1) DuoSet (DY438) and Mouse Leptin DuoSet (DY498) from R&D, as well as oncostatin-M Mouse, ELISA Kit from American Research Products. * Indicates *p*-value < 0.05 [Student's t-test] vehicle versus amlexanox. Error bars presented as standard error from the mean.

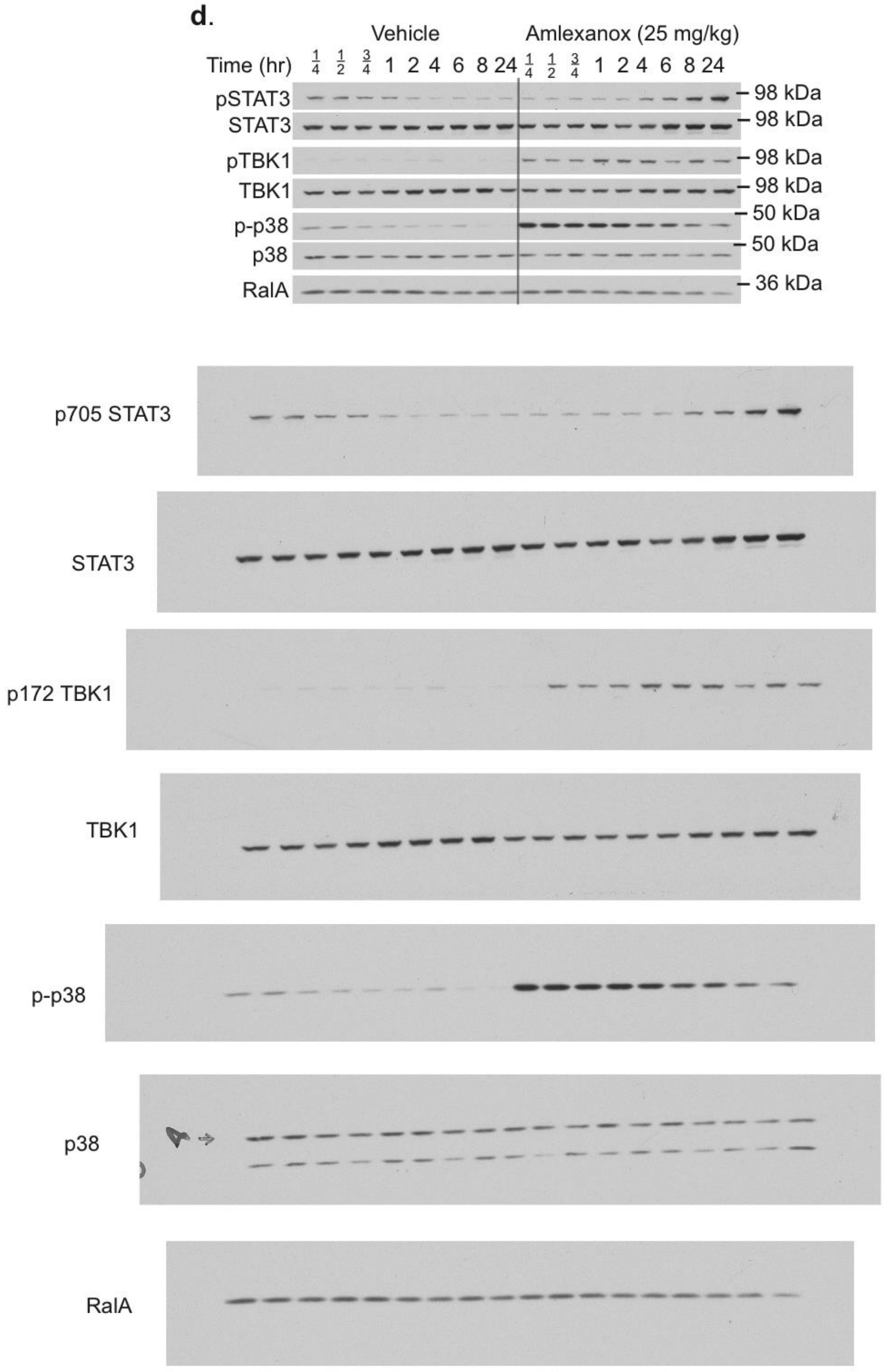

Supplementary Figure 2: **Full western blot scans for Fig. 4d**. The full scans for each blot are shown underneath

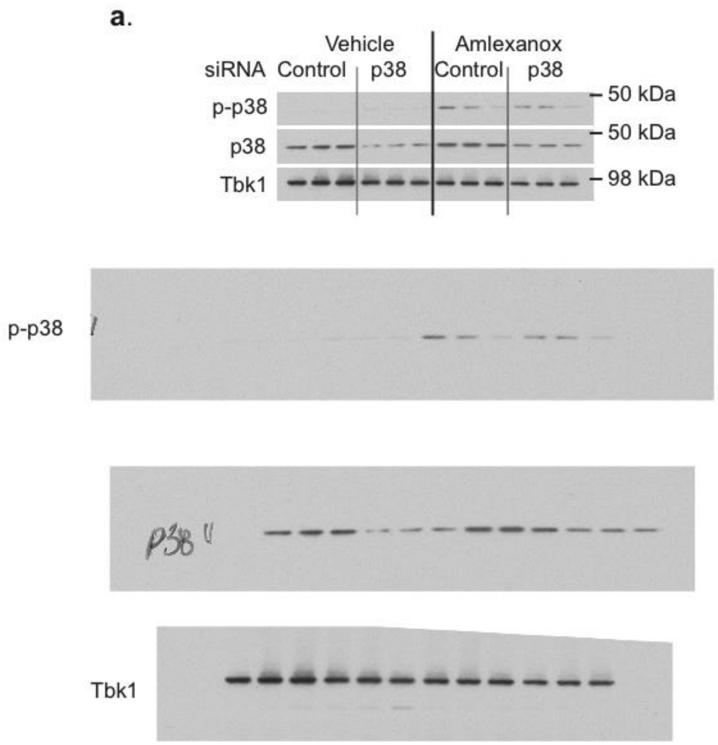

Supplementary Figure 3: **Full western blot scans for Fig. 5a**. The full scans for each blot are shown underneath

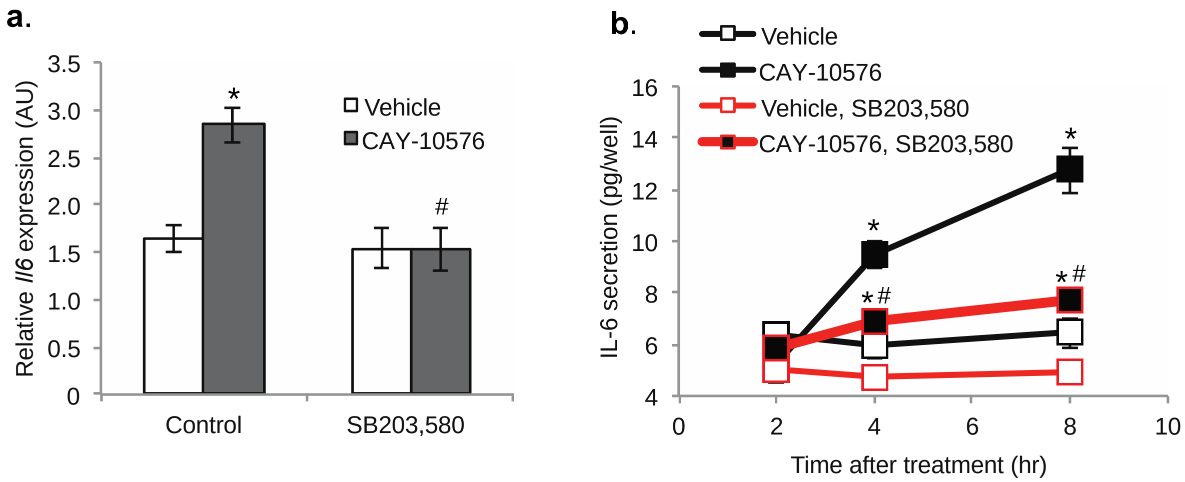

Supplementary Figure 4: **Induction of IL-6 after treatment with CAY-10576**. (**a**) *Il6* expression level in cells treated with 1 μM CAY-10576 for 2 hours after 30 min pretreatment with SB203,580. (**b**) IL-6 secretion into the media. * Indicates *p*-value < 0.05 CAY-10576 versus vehicle. # Indicates *p*-value < 0.05 CAY-10576, SB203,580 versus CAY-10576. Error bars presented as standard error from the mean.

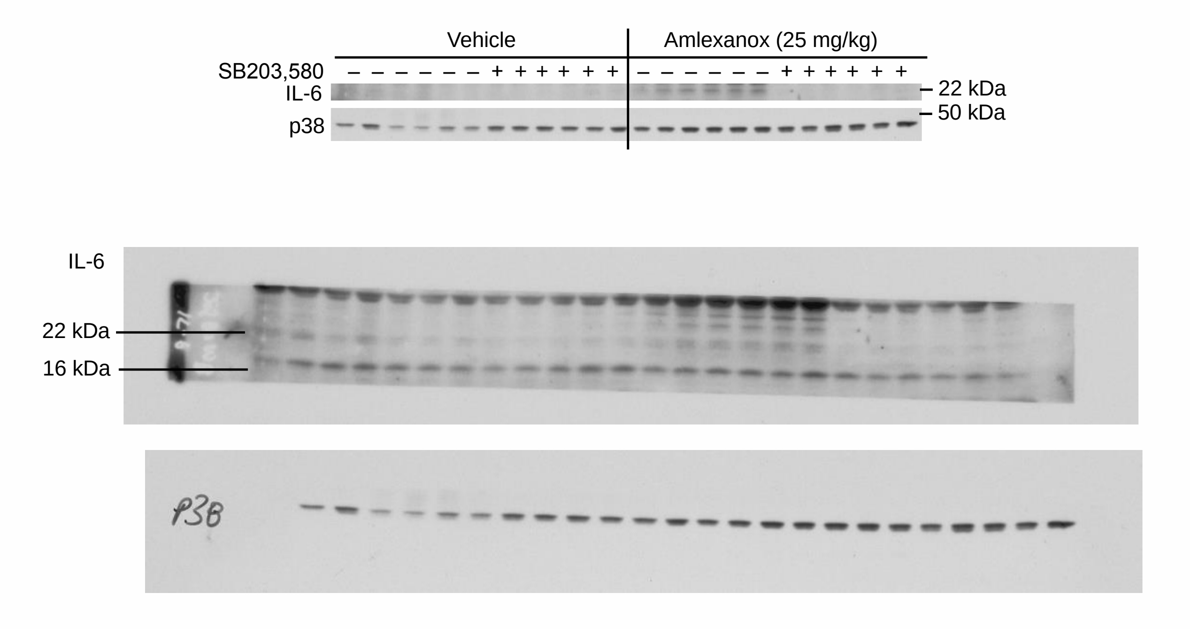

Supplementary Figure 5: **Full western blot scans for Fig. 6d**. The full scans for each blot are shown underneath. Precursor IL-6 (MW 24 kDa) is shown in the main figure. Higher MW species are likely processed forms of IL-6.

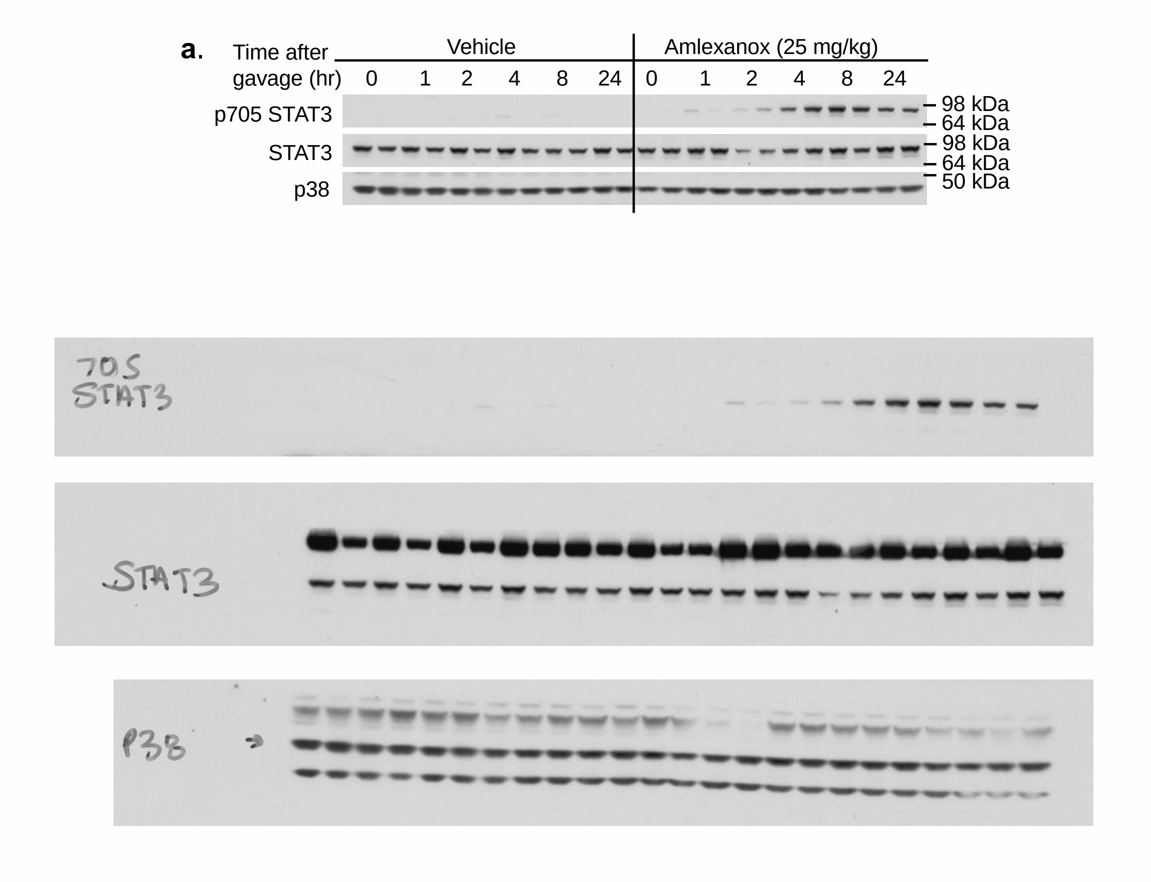

Supplementary Figure 6: **Full western blot scans for Fig. 7a**. The full scans for each blot are shown underneath.

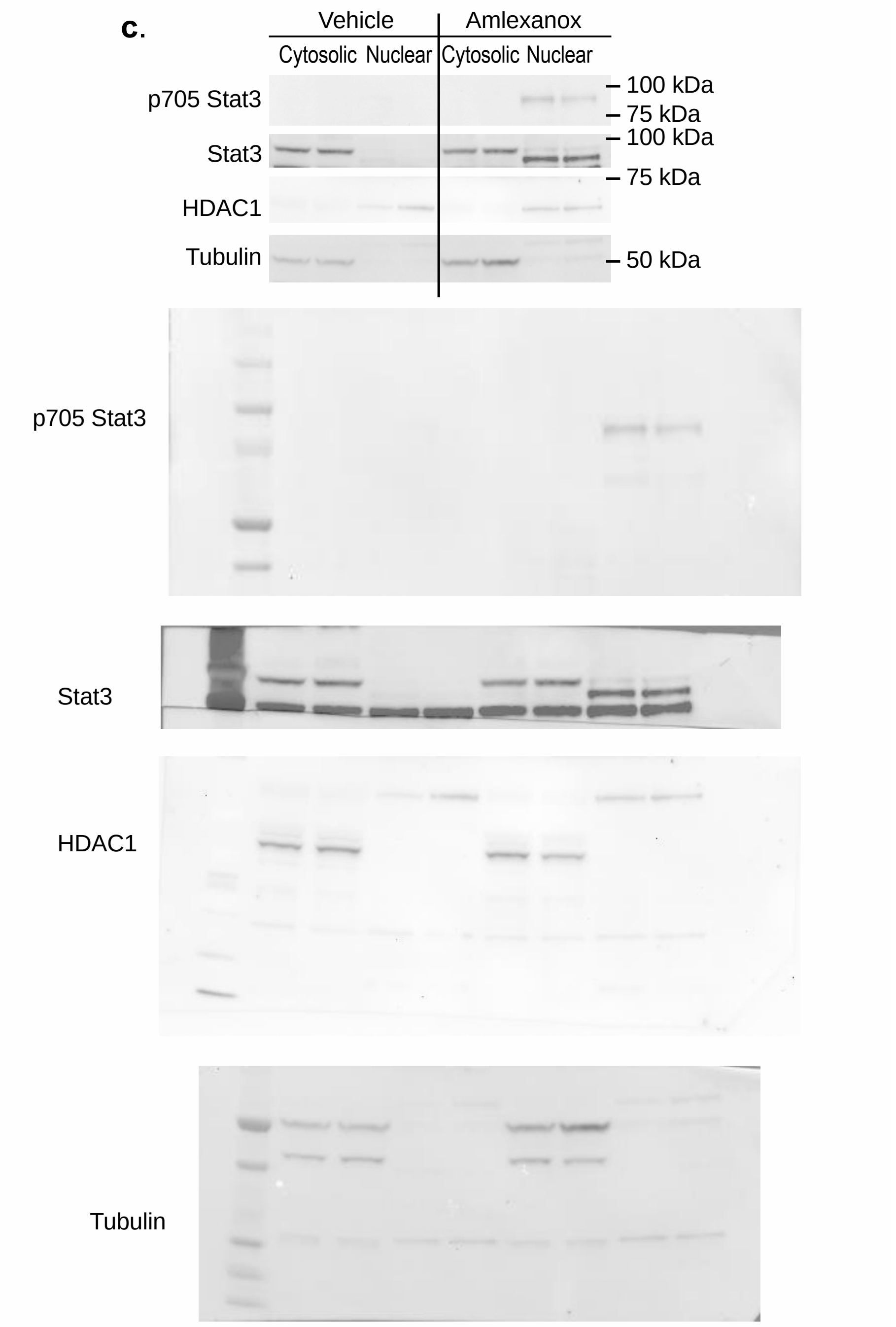

Supplementary Figure 7: **Full western blot scans for Fig. 7c**. The full scans for each blot are shown underneath.

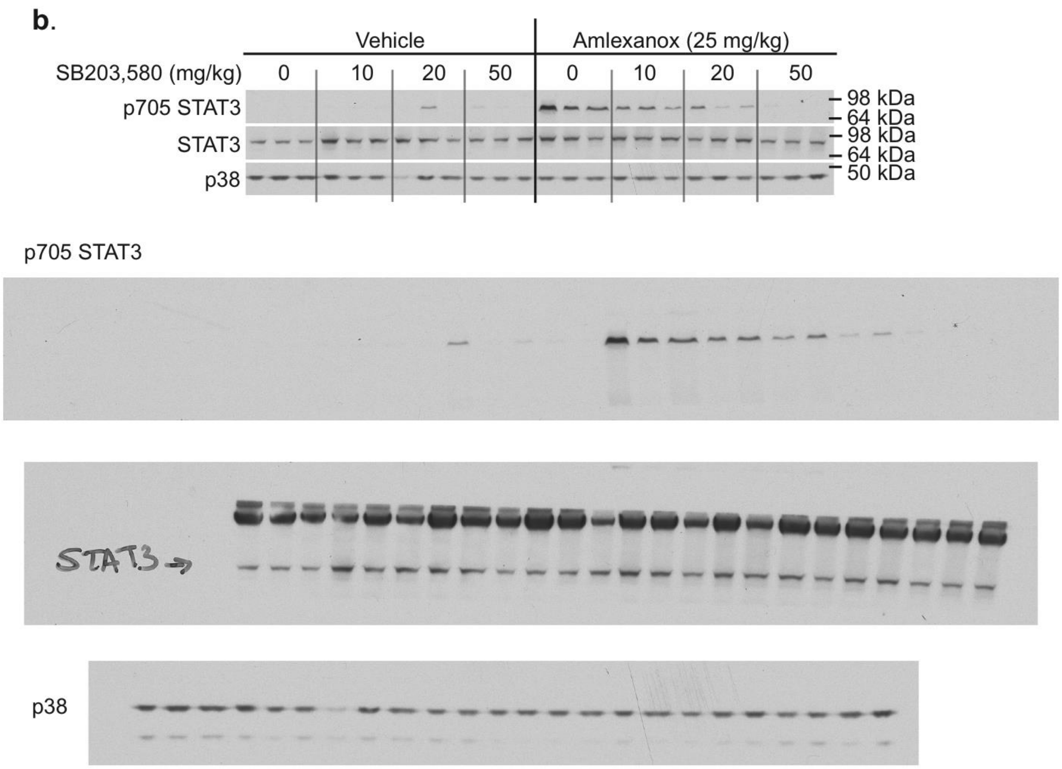

Supplementary Figure 8: **Full western blot scans for Fig. 7d**. The full scans for each blot are shown underneath.

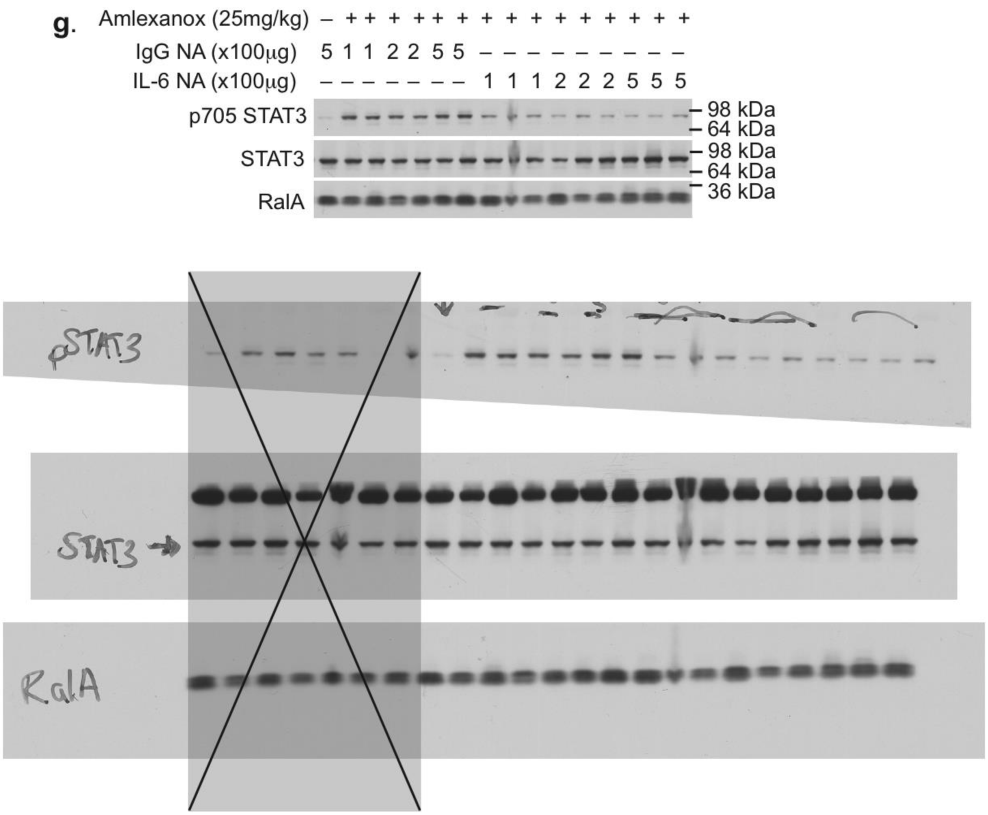

Supplementary Figure 9: **Western blot scans for Fig. 7g**. The full scans for each blot are shown underneath. The excluded section of the gel contains samples from mice treated with neutralizing antibodies intravenously. Marks above pStat3 are permanent marker.

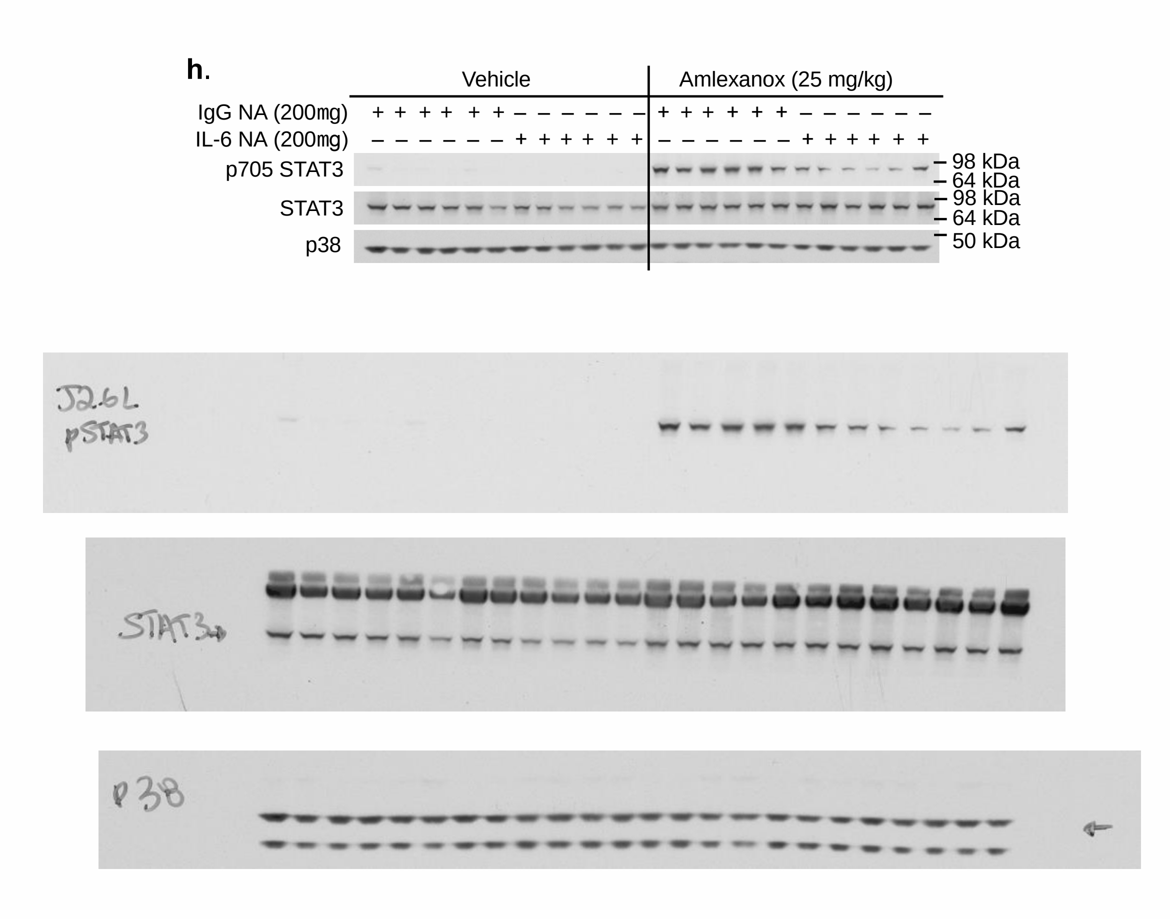

Supplementary Figure 10: **Full western blot scans for Fig. 7h**. The full scans for each blot are shown underneath.

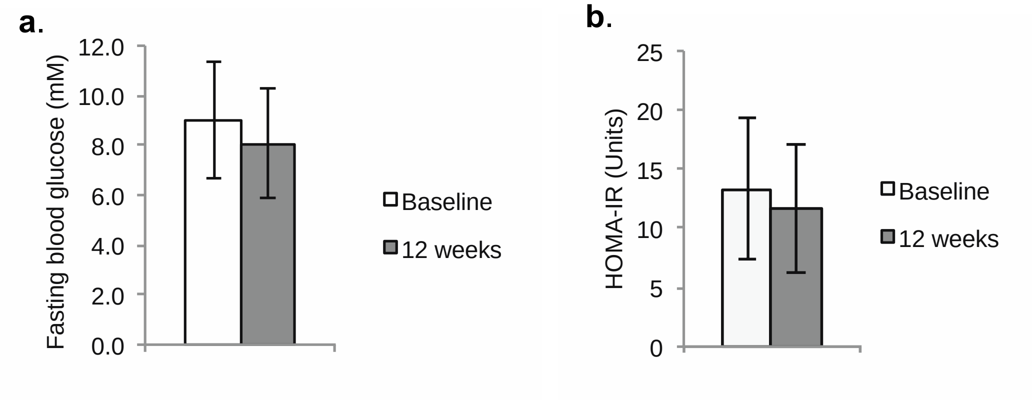

Supplementary Figure 11: **Open label amlexanox trial**. (**a**) Fasting blood glucose levels and (**b**) HOMA-IR score at baseline and after 12 weeks of amlexanox treatment (*n* = 6 patients). Error bars presented as standard error from the mean.

Supplementary Table 1: **KEGG pathway analysis of RNA sequencing data**

Term	Count	%	PValue	Genes
mmu04920:Adipocytokine signaling pathway	15	0.17	0.000001	IRS2, SOCS3, LEPR, RXRG, PRKAB1, PPARGC1A, ADIPOQ, IRS1, STAT3, CAMKK2, LEP, TNFRSF1A, G6PC, CD36, SLC2A1
mmu04630:Jak-STAT signaling pathway	19	0.22	0.000104	IL22RA1, SOCS2, OSMR, CSF2RB2, IL6ST, SOCS3, LEPR, IL4RA, SOCS1, PIM1, CISH, STAT3, SPRY4, LEP, PRLR, PIAS3, SPRED1, JAK3, IL13RA1
mmu04210:Apoptosis	13	0.15	0.000372	BID, IL1R1, CSF2RB2, ATM, IRAK4, TNFRSF1A, IRAK3, TNFSF10, MYD88, CASP9, RIPK1, CASP8, FAS
mmu05200:Pathways in cancer	29	0.33	0.000372	BID, WNT5B, PDGFB, CDH1, ARNT, CASP9, CASP8, SLC2A1, TGFA, RARA, FAS, FH1, FZD8, COL4A1, RXRG, FZD1, BRCA2, SMAD3, DAPK2, FZD5, FZD4, STAT3, CDKN1A, RASSF5, PIAS3, JUN, RASSF1, PDGFRA, PTCH1
mmu04115:p53 signaling pathway	11	0.13	0.000769	BID, CDKN1A, CASP9, BBC3, GADD45G, SERPINE1, CASP8, FAS, CCNG2, GADD45A, ATM
mmu04060:Cytokine-cytokine receptor interaction	21	0.24	0.004945	CXCL1, TNFRSF21, IL1R1, IL22RA1, PDGFB, OSMR, CSF2RB2, IL6ST, LEPR, IL4RA, TNFSF14, IL17RA, CCL6, LEP, INHBB, TNFRSF1A, TNFSF10, PRLR, PDGFRA, FAS, IL13RA1
mmu04010:MAPK signaling pathway	21	0.24	0.011851	IL1R1, PDGFB, MRAS, GNA12, MAP3K6, TNFRSF1A, RPS6KA1, JUN, MAP3K1, MAP3K8, NTRK2, JUND, GADD45G, PDGFRA, RAP1B, FAS, DUSP8, MAP3K13, GADD45A, MAP2K6, DUSP6
mmu00140:Steroid hormone biosynthesis	7	0.08	0.013060	HSD3B2, CYP3A13, CYP7A1, CYP21A1, HSD3B5, SRD5A1, SULT1E1
mmu04930:Type II diabetes mellitus	7	0.08	0.019393	PRKCZ, IRS2, SOCS2, SOCS3, SOCS1, ADIPOQ, IRS1
mmu04620:Toll-like receptor signaling pathway	10	0.11	0.029096	IRAK4, MYD88, JUN, RIPK1, MAP3K8, TICAM1, CASP8, TIRAP, LBP, MAP2K6
mmu04530:Tight junction	12	0.14	0.034407	ACTG1, PRKCZ, OCLN, MPDZ, CGN, MRAS, EPB4.1, CLDN1, MYH9, TJP3, MLLT4, CLDN14
mmu04670:Leukocyte transendothelial migration	11	0.13	0.035579	ACTG1, VCAM1, ICAM1, RASSF5, OCLN, BCAR1, CLDN1, RAP1B, MLLT4, CLDN14, ITGAM
mmu04910:Insulin signaling pathway	12	0.14	0.039459	PRKCZ, G6PC, IRS2, SOCS2, TSC1, SOCS3, SOCS1, FLOT1, PRKAB1, PTPN1, PPARGC1A, IRS1
mmu00982:Drug metabolism	8	0.09	0.045452	CYP2C70, GSTM3, CYP3A13, FMO2, CYP2B9, FMO3, CYP2B13, CYP2A4

Supplementary Table 2: **Q-PCR primer sequences**

Gene	Forward primer	Reverse primer
Adipoq	5’-GCAGGCATCCCAGGACATC-3’	5’-GCGATACATATAAGCGGCTTCT-3’
Arbp	5’-CACTGGTCTAGGACCCGAGAA-3’	5’-AGGGGGAGATGTTCAGCATGT-3’
Emr1	5'-CTGGGATCCTACAGCTGCTC-3'	5'-AGGAGCCTGGTACATTGGTG-3'
G6pc	5’-CGACTCGCTATCTCCAAGTGA-3’	5’-GTTGAACCAGTCTCCGACCA-3’
Il6	5’-TAGTCCTTCCTACCCCAATTTCC-3’	5’-TTGGTCCTTAGCCACTCCTTC-3’
Mapk14	5’-GGCTCGGCACACTGATGAT-3’	5’-TGGGGTTCCAACGAGTCTTAAA-3’
Pdgfra	5’-AGAGTTACACGTTTGAGCTGTC-3’	5’-GTCCCTCCACGGTACTCCT-3’
Socs3	5’-ATGGTCACCCACAGCAAGTTT-3’	5’-TCCACTAGAATCCGCTCTCCT-3’
Tnfa	5'-ACGGCATGGATCTCAAAGAC-3'	5'-AGATAGCAAATCGGCTGACG-3'

Supplementary Table 3: **ChIP primer sequences**

Promoter	Forward primer	Reverse primer
Socs3	5’-CACAGCCTTTCAGTGCAGAG-3’	5’-GGGTATTTACCCGGCCAGT-3’
G6pc (region A)	5’-CAACTCAAACTTGTAAAGAAAAGAAA-3’	5’-TGAATGACCTCCCCCTCCT-3’
G6pc (region B)	5’-CACACACACACACAGAGAGACAG-3’	5’-GCACAATCCTGTATTCCGACT-3’
G6pc (region C)	5’-GTCCCAGTCGGAATACAGGA-3’	5’-TGCCACCTTCTGCTGAGAC-3’
G6pc (region E)	5’-AATGGCGATCAGGCTGTTT-3’	5’-CAATCCAGCCCTGATCTTTG-3’
